# The effect of structured picturebook narrative intervention on emotional understanding in 5–6-year-old Chinese children

**DOI:** 10.3389/fpsyg.2026.1853362

**Published:** 2026-08-18

**Authors:** Mengxue Ni

**Affiliations:** Tianhua College, Shanghai Normal University, Shanghai, China

**Keywords:** cross-cultural evidence, dialogic reading, early childhood intervention, emotional understanding, picturebook narrative, social–emotional learning

## Abstract

This study investigated the effectiveness of an 8-week, structured picturebook narrative intervention on emotional understanding among 5–6-year-old children in eastern China. A total of 97 children were randomly assigned to either an intervention group (*n* = 49) or a control group (*n* = 48). The Test of Emotion Comprehension (TEC) was administered at pre-test and post-test. Repeated-measures ANOVA revealed a significant group×time interaction effect, with the intervention group showing significantly greater improvements in emotion recognition, understanding of emotional causes, and emotional inference (all *p* < 0.001). Between-group effect sizes (Cohen’s d) ranged from 1.12 to 1.62, indicating large intervention effects. These findings confirm that structured, emotion-focused picturebook narrative intervention effectively enhances young children’s emotional understanding, providing culturally relevant evidence for early childhood social–emotional education in the Asia-Pacific region.

## 1 Introduction

Emotional understanding is a core developmental capacity that supports peer interaction, social adaptation, and long-term academic and behavioral adjustment in early childhood (Denham et al., 2012; Jones et al., 2019). A growing body of evidence shows that emotional understanding predicts long-term academic performance, behavioral adjustment, and psychological well-being (Jones et al., 2019).

Dialogic reading is an interactive reading approach where adults use open-ended questions, expansions, and feedback to engage children in the story, fostering language and comprehension skills (Zucker et al., 2025). Interactive or shared reading refers to any reading activity involving shared attention between an adult and child, which may or may not include targeted emotional prompts. While dialogic and interactive book reading have been shown to support emotion-related language and perspective-taking in Western contexts, many classroom practices lack systematic and targeted design for promoting emotional understanding, relying mostly on unstructured reading rather than purposeful interaction. Furthermore, most existing studies have been conducted with Western samples, limiting the cross-cultural generalizability of findings to non-Western contexts such as China.

This study addresses this gap by evaluating an 8-week structured picturebook narrative intervention with a sample of Chinese preschool children. The primary aim is to examine whether a culturally adapted, emotion-focused picturebook intervention improves emotional understanding in 5–6-year-old children, and to provide practical, replicable strategies for early childhood educators in the Asia-Pacific region.

## 2 Literature review

### 2.1 Early childhood emotional understanding: theoretical foundations

Emotional understanding in early childhood is not a single, static skill but a developmental construct that unfolds across multiple interrelated domains (Pons and Harris, 2019). According to the *Test of Emotion Comprehension (TEC)* framework, which guided the present study, children’s emotional understanding progresses through three hierarchical levels:

1) *Recognition of emotions*: The ability to identify basic emotional expressions (e.g., happy, sad, angry, scared) in facial cues and situational contexts. This foundational skill typically develops by age 3–4, as children learn to link visual cues to internal affective states.
2) *Understanding of emotional causes*: The ability to explain why events, actions, or social interactions trigger specific emotions. For example, children gradually understand that receiving a gift causes happiness, while losing a toy causes sadness. This skill requires children to connect external events to internal mental states, and it is strongly supported by adult scaffolding and dialogic interaction (Denham et al., 2012).
3) *Emotional inference and regulation*: The ability to predict others’ emotions based on context and to use emotional knowledge to guide behavior. This includes perspective-taking (e.g., “My friend will feel sad if I do not share”) and understanding that emotions can change over time or be hidden.

This developmental sequence is shaped by both biological maturation and social experiences, with early parent–child and teacher–child interactions playing a critical role (Tang et al., 2018). In particular, shared book reading with intentional emotional talk has been identified as a powerful context for fostering these skills, as it provides children with repeated opportunities to explore emotions in low-stakes, narrative scenarios (Alvarenga et al., 2020).

### 2.2 Picturebook narrative and emotional learning

Picturebooks provide developmentally appropriate, visual contexts for children to explore abstract emotional concepts. Emotion-focused dialogic reading, which includes targeted prompts about characters’ feelings, causes, and consequences, has been shown to strengthen children’s emotion vocabulary, perspective-taking, and affective reasoning (Alvarenga et al., 2020; Grazzani and Ornaghi, 2021). Despite these benefits, rigorous evidence from Asian cultural contexts remains limited. Cultural differences in emotional expression norms and classroom practices may influence how children respond to emotion-focused interventions, highlighting the need for locally validated research (Sun et al., 2021).

## 3 Methods

### 3.1 Participants

Participants were 97 typically developing children aged 5–6 years recruited from two public kindergartens in eastern China. Intervention group: 49 children (24 boys, 25 girls; mean age = 5.42 years, SD = 0.38). Control group: 48 children (23 boys, 25 girls; mean age = 5.39 years, SD = 0.41). All children spoke Mandarin as their primary language and had no reported developmental delays or behavioral disorders. At baseline, there were no significant differences between groups in age, gender, or pre-test emotional understanding scores (all *p* > 0.05).

### 3.2 Ethics statement

This study was conducted in accordance with the Declaration of Helsinki and approved by the Ethics Committee of Shanghai Normal University Tianhua College. Written informed consent was obtained from all parents or legal guardians prior to data collection, and verbal assent was obtained from each child. No identifiable personal information was collected or stored; all data were anonymized using unique participant codes. Pre-test and post-test responses were linked via these codes to ensure confidentiality.

### 3.3 Measures

The Chinese version of the Test of Emotion Comprehension (TEC) was used to assess emotional understanding (adapted from Pons and Harris, 2019). The scale includes three subscales: emotion recognition, understanding of emotional causes, and emotional inference. The scale demonstrated good internal consistency in this sample (Cronbach’s *α* = 0.82). The full scale and scoring rubric are provided in Appendix A.

### 3.4 Intervention procedure

The intervention group received an 8-week structured picturebook narrative intervention, delivered twice weekly in 20-min small-group sessions (4–5 children per group) by two trained research assistants. The research assistants held master’s degrees in early childhood education and completed 10 h of training on the intervention protocol, including scripted prompts and facilitation strategies.

#### 3.4.1 Intervention materials

A set of 16 culturally relevant, emotion-focused picturebooks was selected, covering basic emotions (happiness, sadness, anger, fear) and social emotions (empathy, shame, pride). The books were chosen based on alignment with Chinese cultural contexts, clear emotional cues, and developmentally appropriate themes for 5–6-year-olds. All selected books have been translated into Chinese and are widely used in Chinese kindergartens; the research assistants adapted the discussion questions to focus on culturally familiar scenarios (e.g., family relationships, school friendships, Chinese festivals) while keeping the emotional content unchanged. The full list is presented in Appendix B.

#### 3.4.2 Session structure

Each 20-min session followed a standardized sequence:

1) Opening (3 min): Warm-up activity to activate emotion vocabulary (e.g., “Today we are reading a story about feelings. How do you feel today?”).
2) Dialogic reading (10 min): The research assistant read the picturebook aloud, using four types of targeted emotional prompts:

*Labeling*: Naming characters’ emotions (“Look, the rabbit’s ears are down. How do you think the rabbit feels?”).

1) *Cause prompts*: Asking children to explain the source of emotions (“Why do you think the rabbit is sad? Did something happen?”).
2) *Inference prompts*: Asking children to predict future emotions (“The rabbit’s friend is coming to play. How will the rabbit feel then?”).
3) *Perspective-taking prompts*: Asking children to relate the story to their own lives (“Have you ever felt like the rabbit? What did you do?”).
4) Activity extension (5 min): Role-play, drawing, or sharing to reinforce emotional understanding. For example, after reading a story about anger, children might draw a picture of a time they felt angry and share how they calmed down.
5) Closing (2 min): Summary of key emotions and vocabulary (“Today we learned about feeling sad and what makes people sad. We can help our friends when they feel sad too!”).

The control group received routine shared reading sessions (twice weekly, 20 min) with the same picture books as the intervention group. These sessions were delivered by the same two trained research assistants to ensure consistency across groups. However, unlike the intervention sessions, control group reading did not include targeted emotional prompts, structured discussion, or activity extensions. The research assistants read the stories aloud, answered children’s basic questions about the plot, but did not guide children to analyze characters’ emotions, causes, or perspectives. This design ensures that the only systematic difference between groups was the emotion-focused dialogic reading component, allowing us to isolate its effect on emotional understanding.

Each standardized session contained exactly eight emotion-focused guiding questions evenly divided into labeling, cause, inference, and perspective-taking prompts. All reading activities were uniformly limited to 20 min, with identical four-stage segment time allocation across every session. Children were divided into fixed small groups of four to five participants for the full 8-week intervention. To maintain consistent interaction quality, research assistants followed a unified feedback framework: when children provided incorrect or irrelevant emotional responses, assistants used scaffolding auxiliary questions to guide self-reflection rather than directly correcting or denying children’s answers. This standardized feedback strategy was fully covered in pre-intervention training and verified through session recording fidelity checks.

#### 3.4.3 Research assistant training, calibration, and implementation fidelity

The 10-h training covered early childhood emotional development theories, dialogic reading skills, standardized emotional questioning, and practice of common interaction scenarios. Facilitator calibration included unified lectures and simulated teaching sessions. After practice, all research assistants used the same prompt scripts to reduce individual differences. Implementation fidelity was ensured by recording all sessions and randomly checking 25% of them. The overall fidelity rate reached 92%, indicating the intervention was implemented as planned.

### 3.5 Data analysis

All analyses were conducted using SPSS 26.0. Repeated-measures ANOVA was used to examine the group × time interaction effect on emotional understanding scores, with age and gender included as covariates. Independent-samples t-tests were used to compare post-test scores between groups. Cohen’s d was calculated to report between-group effect sizes, with values of 0.2, 0.5, and 0.8 interpreted as small, medium, and large effects, respectively. Statistical significance was set at *p* < 0.05.

## 4 Results

### 4.1 Baseline equivalence

At pre-test, there were no significant differences between the intervention and control groups on total emotional understanding scores or any subscale (all *p* > 0.05). Age and gender were not significantly correlated with pre-test scores (*p* > 0.05).

### 4.2 Repeated-measures ANOVA results

Repeated-measures ANOVA revealed a significant main effect of time [*F*(1,93) = 128.47, *p* < 0.001, η^2^ = 0.58] and a significant group × time interaction effect [F(1,93) = 96.23, *p* < 0.001, η^2^ = 0.51], indicating that the intervention group improved significantly more than the control group.

### 4.3 Post-test comparisons

As shown in Table 1, the intervention group scored significantly higher than the control group on all subscales and the total emotional understanding score at post-test (all *p* < 0.001). Effect sizes (Cohen’s d) ranged from 1.12 to 1.31, indicating large intervention effects.

**Table 1:** Pre-test and post-test emotional understanding scores by group.

Measure	Intervention (*n* = 49) mean (SD)	Control (*n* = 48) mean (SD)	*t* (95)	*p*-value	Cohen’s d
Pre-test Total	32.14 (4.21)	31.89 (4.07)	0.29	0.77	0.06
Post-test Total	43.82 (3.98)	37.24 (4.21)	8.12	<0.001	1.62
Emotion Recognition	15.46 (1.79)	13.08 (1.94)	6.21	<0.001	1.28
Emotional Causes	14.62 (1.88)	12.37 (2.05)	5.79	<0.001	1.15
Emotional Inference	13.74 (1.69)	11.79 (1.81)	5.43	<0.001	1.12

Subscale score ranges are 0–16; total score range is 0–56.

## 5 Discussion

### 5.1 Effects of the structured picture book narrative intervention

The results confirm that an 8-week structured picture book narrative intervention significantly improves emotional understanding in 5–6-year-old Chinese children. The large effect sizes across all subscales suggest that targeted, emotion-focused dialogic reading is an effective strategy for enhancing children’s ability to recognize, explain, and infer emotions. These findings align with previous research showing that structured emotional prompts during shared reading strengthen children’s affective reasoning (Alvarenga et al., 2020; Ornaghi et al., 2018).

All 16 selected picturebooks were explicit emotion-oriented books, which directly introduce emotions, emotion vocabulary, and emotion-related skills, without pure narrative books where emotions are only implied in the plot. The high proportion of explicit emotional books helped children quickly identify and understand emotions, which is an important reason for the significant intervention effect. The clear emotional cues in these books matched the structured dialogic prompts, making it easier for children to connect story content with emotional understanding.

### 5.2 Cross-cultural context and practical implications for early childhood educators

Beyond classroom operation guidance, this structured picturebook narrative intervention delivers clear policy implications for early childhood social–emotional education in China. Current national kindergarten curriculum guidelines attach growing importance to children’s emotional development, yet most kindergartens lack systematic, replicable emotional reading frameworks and rely on informal, unstructured story sharing. The manualized 8-week intervention proposed in this study matches the policy demand for standardized social–emotional learning courses for 5–6-year-olds. Local education departments can integrate this dialogic reading protocol into pre-service and in-service training for preschool teachers, forming a culturally compatible emotional education module that fits Chinese family and campus cultural contexts. For regional early childhood education policy makers, the large intervention effect sizes reported in this research provide empirical evidence to support the popularization of emotion-centered shared reading in public kindergartens across East Asia.

This study extends the predominantly Western literature on picture book interventions by providing empirical evidence from a Chinese preschool context. While emotion-focused dialogic reading has been widely studied in Western settings, its effectiveness in Asian contexts remains under documented, despite cultural differences in emotional expression norms and classroom practices (Sun et al., 2021). The large effect sizes observed in this study suggest that structured picture book interventions are highly adaptable to Chinese preschool settings, offering three key, actionable implications for educators.

In traditional Chinese early childhood education, more attention has been paid to behavioral norms and academic preparation, while systematic emotional discussion and expression are relatively less emphasized. Children are often encouraged to control overt emotions in public situations. This intervention adopted explicit emotional dialogue activities, which not only followed the existing emotional education ideas in Chinese kindergartens but also made innovations by adding systematic emotional questioning and discussion. By combining picture books with familiar cultural themes such as family and friendship, the intervention was consistent with Chinese cultural values, which helped children engage deeply and promoted the improvement of emotional understanding.

#### 5.2.1 Implement structured, emotion-focused dialogic reading instead of passive story time

Rather than simply reading books aloud, educators should use targeted emotional prompts to guide children’s thinking. For example, after reading a story about a character who feels sad, educators can ask:

1) “Why do you think the character felt sad?” (Cause-focused prompt)
2) “How do you think the character will feel when their friend helps them?” (Inference prompt)
3) “How would you feel if you were in the character’s place?” (Perspective-taking prompt)

These prompts encourage children to move beyond passive listening to actively analyze and reflect on emotions.

#### 5.2.2 Select culturally relevant picturebooks that reflect children’s daily experiences

The intervention in this study used picture books with themes of family relationships, peer cooperation, and everyday social interactions, which are highly familiar to Chinese children. Educators are encouraged to choose books that align with their students’ cultural contexts, as this increases children’s engagement and helps them connect story emotions to their own lives. For example, stories about grandparents, school friendships, or traditional festivals can provide meaningful opportunities for emotional learning.

#### 5.2.3 Integrate short, structured emotional activities into daily routines

To reinforce learning, educators can pair picture book reading with brief, hands-on activities, such as:

1) Drawing a picture of a time they felt the same emotion as the story character
2) Role-playing the story’s emotional scenes with puppets or peers
3) Creating an “emotion chart” to track how characters feel throughout the story

These activities help children translate abstract emotional concepts into concrete, personal experiences.

Together, these strategies can help early childhood educator’s move beyond ad-hoc emotional discussions to implement evidence-based, culturally responsive social–emotional learning in their classrooms.

### 5.3 Limitations and future directions

Limitations include a restricted age range, urban-only sample, lack of long-term follow-up, and reliance on child-reported measures. Future research may include longitudinal tracking, broader age and regional samples, and multi-informant assessments.

## 6 Conclusion

An 8-week structured picture book narrative intervention significantly improves emotion recognition, understanding of emotional causes, and emotional inference among 5–6-year-old Chinese children. This developmentally appropriate, culturally adapted intervention is feasible for widespread implementation in kindergarten settings and provides an evidence-based strategy for enhancing early childhood social–emotional education in the Asia-Pacific region.

## Data Availability

The raw data supporting the conclusions of this article will be made available by the authors, without undue reservation.
