# Supplementary material for: The effect of structured picturebook narrative intervention on emotional understanding in 5–6-year-old Chinese children
Source: Front Psychol. 2026 Aug 18;17:1853362. doi: 10.3389/fpsyg.2026.1853362 (PMC13529665; doi:10.3389/fpsyg.2026.1853362)
Supplement: Supplementary file 1 [file Supplementary_file_1.docx]

# Appendix A: Test of Emotion Comprehension (TEC)

The Test of Emotion Comprehension (TEC) was used to assess three dimensions of emotional understanding:

1. **Emotion recognition**: Identifying facial expressions of happiness, sadness, anger, and fear.
2. **Understanding of emotional causes**: Explaining why a character feels a certain emotion based on situational cues.
3. **Emotional inference**: Predicting characters’ emotions based on story contexts.

The scale includes 16 items. Total score ranges from 0 to 56 (each item scored 0–3 based on accuracy). Higher scores indicate better emotional understanding. In this study, Cronbach’s α = 0.82, indicating good internal consistency.
