# Supplementary material for: The effect of structured picturebook narrative intervention on emotional understanding in 5–6-year-old Chinese children
Source: Front Psychol. 2026 Aug 18;17:1853362. doi: 10.3389/fpsyg.2026.1853362 (PMC13529665; doi:10.3389/fpsyg.2026.1853362)
Supplement: Supplementary file 2 [file Supplementary_file_2.docx]

# Appendix B: List of 16 Picturebooks Used in the Intervention

Note. All books have been translated into Chinese and are widely used in Chinese kindergartens.

1) Cain, J. (2000). The way I feel. Parenting Press. Emotions: Frustration, shyness, jealousy, pride, sadness, anger, excitement.
2) Curtis, J. L. (1998). Today I feel silly & other moods that make my day. HarperCollins. Emotions: Silly, angry, excited, grumpy, sad.
3) Spelman, C. M. (2000). When I feel angry. Albert Whitman & Company. Emotion: Anger and anger management.
4) Lang, S. (2018). Grumpy monkey. Random House. Emotion: Grumpiness and emotional acceptance.
5) Spelman, C. M. (2002). When I feel scared. Albert Whitman & Company. Emotion: Fear and coping.
6) Percival, T. (2019). Ruby finds a worry. Bloomsbury Children's Books. Emotion: Worry and emotional disclosure.
7) Spelman, C. M. (2002). When I feel sad. Albert Whitman & Company. Emotion: Sadness and coping.
8) Herman, S. (2019). The sad dragon. DG Books Publishing. Emotion: Grief and loss.
9) Doerrfeld, C. (2018). The rabbit listened. Dial Books for Young Readers. Emotion: Empathy, kindness, active listening.
10) Spelman, C. M. (2018). When I care about others. Albert Whitman & Company. Emotion: Empathy and perspective-taking.
11) Karst, P. (2000). The invisible string. DeVorss Publications. Emotion: Love and connection.
12) Lee, B. W. (2019). The boy with big, big feelings. Beaming Books. Emotion: Emotional awareness, acceptance, self-expression.
13) Dr. Seuss. (1996). My many colored days. Alfred A. Knopf. Emotion: Color-emotion association, mood variation.
14) Garcia, G. (2017). Listening to my body. Take Heart Press. Emotion: Interoception – connecting physical sensations to emotions.
15) Llenas, A. (2015). The color monster: A pop-up book of feelings. New York, NY: Little, Brown and Company. Emotion: Happiness, sadness, anger, fear, and calm.
16) Parr, T. (2000). The feelings book. Little, Brown and Company. Emotion: Complete range of feelings and emotional expression.
